# Identification of Membrane-Bound Lytic Murein Transglycosylase A (MltA) as a Growth Factor for *Francisella novicida* in a Silkworm Infection Model

**DOI:** 10.3389/fcimb.2020.581864

**Published:** 2021-01-22

**Authors:** Takemasa Nakamura, Takashi Shimizu, Fumiya Inagaki, Shoma Okazaki, Shib Shankar Saha, Akihiko Uda, Kenta Watanabe, Masahisa Watarai

**Affiliations:** ^1^ Laboratory of Veterinary Public Health, Joint Faculty of Veterinary Medicine, Yamaguchi University, Yamaguchi, Japan; ^2^ Department of Pathology and Parasitology, Patuakhali Science and Technology University, Barisal, Bangladesh; ^3^ Department of Veterinary Science, National Institute of Infectious Diseases, Tokyo, Japan

**Keywords:** *Francisella*, tularemia, silkworm, transglycosylase, infection model

## Abstract

*Francisella tularensis*, the causative agent of tularemia, is transmitted by arthropod vectors within mammalian hosts. The detailed mechanisms contributing to growth and survival of *Francisella* within arthropod remain poorly understood. To identify novel factors supporting growth and survival of *Francisella* within arthropods, a transposon mutant library of *F. tularensis* subsp. *novicida* (*F. novicida*) was screened using an *F. novicida*–silkworm infection model. Among 750 transposon mutants screened, the *mltA*-encoding membrane-bound lytic murein transglycosylase A (MltA) was identified as a novel growth factor of *F. novicida* in silkworms. Silkworms infection with an *mltA* deletion mutant (Δ*mltA*) resulted in a reduction in the number of bacteria and prolonged survival. The Δ*mltA* strain exhibited limited intracellular growth and cytotoxicity in BmN4 silkworm ovary cells. Moreover, the Δ*mltA* strain induced higher expression of the antimicrobial peptide in silkworms compared to the wild-type strain. These results suggest that *F. novicida* MltA contributes to the survival of *F. novicida* in silkworms *via* immune suppression-related mechanisms. Intracellular growth of the Δ*mltA* strain was also reduced in human monocyte THP-1 cells. These results also suggest the contribution of MltA to pathogenicity in humans and utility of the *F. novicida*–silkworm infection model to explore *Francisella* infection.

## Introduction

*Francisella tularensis* is a facultative intracellular pathogen and the causative agent of tularemia (Ellis et al., 2002). It shows high infectivity *via* aerosol transmission; as few as 10 F*. tularensis* bacterial cells can cause disease in humans (McLendon et al., 2006). As such, *F. tularensis* may be deployed as a potential biological weapon and is currently classified as a category A bioterrorism agent by the US Centers for Disease Control and Prevention (CDC) (Dennis et al., 2001). Among the different subspecies, only *F. tularensis* subsp. *tularensis* (Type A) and *F. tularensis* subsp. *holarctica* (Type B) are highly pathogenic to humans (Maurin, 2015). *F. tularensis* subsp. *novicida*, referred to here as *F. novicida*, shares significant homology with *F. tularensis* subsp. *tularensis*, as it is virulent in mice but nonpathogenic to immunocompetent humans. As such, *F. novicida* serves as a surrogate organism for experimental studies (Kingry and Petersen, 2014). Although *F. tularensis* has been identified in a wide range of organisms, including mammals, birds, amphibians, and invertebrates (Keim et al., 2007), the disease tularemia predominantly occurs in rodents, voles, hares, rabbits, and humans (Carvalho et al., 2014). Interactions between *F. tularensis* and arthropod hosts play major roles in supporting its life cycle (Petersen et al., 2009). The pathogen is transmitted between animals including humans by arthropod vectors such as deer flies, horse flies, ticks, and mosquitoes (Carvalho et al., 2014). The routes of infection to humans are varied, e.g. inhalation, contact with infection animals or contaminated tissues (Ellis et al., 2002). Arthropods vectors are also considered as one of main infection routes of *F. tularensis* (Carvalho et al., 2014). However, the detailed mechanisms of the growth and survival of *F. tularensis* in arthropod vectors remain poorly understood.

Mammalian infection models are typically used for the identification and evaluation of virulence factors; however, large-scale experiments using mammals raise the issue of ethics and feasibility. To address this issue, we have developed a non-mammalian infection model using the silkworm, *Bombyx mori*. As silkworms have been domestically cultivated for the past 5000 years to produce silk, there are many lineages, which are well-defined and maintained, and tools for genetic engineering of these organisms are also readily available. Silkworms survive at 37°C; this is the optimal temperature for humans and one that supports the growth of most pathogens (Kaito et al., 2011). The body size of silkworm larva (3–85 mm) and the period of the larvae (25 days) facilitate injection of pathogens and experimental repetitions. Therefore, *B. mori* larvae have been used as infection models for experiments focused on a large variety of agents, such as bacteria, viruses, and fungi (Kaito et al., 2002; Jiang et al., 2016; Kaito, 2016; Uchida et al., 2016). Recently, non-mammalian infection models, including the wax moth (*Galleria mellonella*), fruit fly (*Drosophila melanogaster*), cockroach (*Blaptica dubia*), and zebrafish (*Danio rerio*), have all been employed as models to promote understanding of host–pathogen interactions associated with *Francisella* (Aperis et al., 2007; Vonkavaara et al., 2008; Vojtech et al., 2009; Ahlund et al., 2010; Moule et al., 2010; Eklund et al., 2017). Compared with these infection models, the silkworm model has advantages with respect to body size, breeding period, breeding equipment, temperature, cost, and availability of genetic information. We have developed both symbiotic and pathogenic silkworm infection models. Infection with *F. tularensis* subsp. *holarctica* LVS (LVS), the bacteria fail to proliferate and are maintained in the silkworm in a symbiotic relationship (Suzuki et al., 2016). By contrast, *F. novicida* proliferates in silkworms and promotes a lethal infection; these results underscore the pathogenic properties of *F. novicida* in silkworm (Saha et al., 2017).

We herein carried out a large-scale screening of a *F. novicida* transposon mutant library using the *F. novicida*–silkworm infection model. Membrane-bound lytic murein transglycosylase A (MltA) was identified as a novel factor, which supports growth and survival of *Francisella* in arthropod species.

## Materials and Methods

### Bacterial Strains and Culture Conditions

*F. novicida* U112 was obtained from the Pathogenic Microorganism Genetic Resource Stock Center (Gifu University, Japan). *F. novicida* was cultured aerobically at 37°C in a chemically defined medium (CDM) (Nagle et al., 1960), brain heart infusion broth (Becton, Dickinson and Company, Franklin Lakes, NJ) supplemented with cysteine (BHIc) (Mc Gann et al., 2010), or BHIc containing 1.5% agar (Wako Laboratory Chemicals, Osaka, Japan). All experiments were conducted in compliance with the institutional biosecurity guidelines and were approved by Yamaguchi University.

### Cell Culture

Human monocytic THP-1 cells were grown in Roswell Park Memorial Institute (RPMI) 1640 medium (Sigma-Aldrich, St. Louis, MO) supplemented with 10% heat-inactivated fetal bovine serum at 37°C in an atmosphere containing 5% CO_2_. Silkworm ovary-derived BmN4 cells were grown in Sf-900 III SFM medium (Thermo Fisher, Waltham, MA) at 27°C.

### Silkworms

Fourth instar *B*. *mori* larvae (Hu/Yo × Tukuba/Ne) were obtained from Ehime-Sanshu (Ehime, Japan). The larvae were raised by feeding them with Silkmate 2M (Nosan Corporation, Kanagawa, Japan) at room temperature (25°C).

### Plasmid Construction, Transformation, and Transfection

**Supplementary Table 1** lists the primer sets and templates used to construct the plasmids used in this study. Polymerase chain reaction (PCR) was performed using KOD-Plus-Neo polymerase (Toyobo, Osaka, Japan), and ligation was performed using an In-Fusion HD Cloning Kit (Takara Bio, Shiga, Japan). Plasmids were used to transform *F. novicida via* electroporation. Specifically, bacterial cells were suspended 0.5 M sucrose with 2 μg of plasmid DNA and were electroporated using a Bio-Rad micropulser (Bio-Rad, Hercules, CA) at 3.0 kV, 10 µF, and 600 Ω with 0.2 cm cuvette; transformants were precultured in CDM medium overnight. To select the transformed bacteria, the pre-incubated bacteria were cultured on BHIc agar plates containing 30 μg/ml kanamycin or 2.5 μg/ml chloramphenicol.

### Construction of a Transposon Mutant Library

The transposon mutant library was constructed using the Ez-Tn5 transposon system (Epicentre, Madison, WI) as previously reported (Nakamura et al., 2019). Briefly, the multiple cloning site of pMOD3 was opened by digestion with Hind III and EcoRI, and the kanamycin resistance cassette of pKEK1140 (Rodriguez et al., 2008) was ligated into these sites to generate pMOD3-FtKm. The transposon moiety of pMOD3-FtKm was amplified by PCR, purified, mixed with transposase according to the instruction manual, and then used to transform *F. novicida via* electroporation. Transformed bacteria were cultured on BHIc plates containing 30 μg/ml kanamycin.

### Sequence Analysis of Transposon Mutants

pMOD3 harbors the *E. coli* R6Kγ origin of replication. The genomes of *F*. *novicida* transposon mutants were purified using a PureLink Genomic DNA Mini Kit (Thermo Fisher) and digested with a combination of restriction enzymes, such as XhoI, BglII, EcoRI, SalI, NotI, and BamHI. The ends of the digested DNAs were then blunted using a DNA Blunting Kit (Takara Bio) and ligated using Ligation High Ver. 2 (Toyobo). The ligated DNA was used to transform One Shot PIR1 Chemically Competent *E. coli* (Thermo Fisher). The transformed *E. coli* were selected for kanamycin resistance, and the plasmid DNAs were purified. Sequence analysis was performed using the primer described in the instruction manual for the Ez-Tn5 transposon system.

### Construction of *F. novicida* Mutants

A deletion mutant of *dotU* (Δ*dotU*) was previously constructed (Shimizu et al., 2019) through group II intron insertion using a TargeTron Gene Knockout System (Sigma-Aldrich), which was modified for *Francisella* species (Rodriguez et al., 2008). A deletion mutant of *mltA* (Δ*mltA*) was generated *via* homologous recombination using the *Francisella* suicide vector pFRSU (Shimizu et al., 2019). The upstream and downstream regions of *mltA* (1.5 kbp each) were cloned into the BamHI site of pFRSU to generate pFRSU-mltA. The pFRSU-mltA vector (2 μg) was used to transform *F. novicida*; transformants were selected on BHIc plates containing 30 μg/ml kanamycin. Isolated bacteria were cultured in BHIc without antibiotics overnight and then plated on BHIc plates containing 5% sucrose. The deletion of the *mltA* gene was confirmed *via* PCR.

### Green Fluorescent Protein (GFP)- and MltA-Expressing *F. novicida* Strains

A GFP-expressing plasmid pOM5-GFP was constructed according to published procedures (Shimizu et al., 2019). The *F. novicida* chromosomal *mltA* gene with its native promoter region (300 bp upstream) was cloned into pOM5 to generate pOM5-MltA. pOM5-MltA and pOM5-GFP were used to transform the wild-type or Δ*mltA* strains of *F. novicida via* electroporation.

### Silkworm Infection Assay

Hemocoels of day 2 fifth instar larvae were inoculated with 50 μl of bacterial suspension in BHIc medium at 5 × 10^6^ colony-forming unit (CFU)/larva using a 1 ml syringe equipped with a 30-gauge needle (Terumo Corp., Tokyo, Japan). After inoculation, the silkworms were incubated at room temperature with free access to food. The mortality rate of the infected silkworms was assessed daily for 9 days. The bacterial load was evaluated at the same time each day for 4 days after infection. To obtain bacterial counts (CFU/larva) from whole silkworm bodies, the infected larvae were placed in disposable 15 ml centrifuge tubes and homogenized in 3 ml phosphate-buffered saline (PBS) using Biomasher SP (Funakoshi Co., Ltd, Tokyo, Japan) followed by centrifugation at 300 × g for 30 s. Appropriate dilutions of the suspension samples were spread onto BHIc agar plates to facilitate colony counts.

### Intracellular Growth Assay

THP-1 cells (4 × 10^5^ cells/well) were incubated in a 48-well tissue culture plate with 200 nM phorbol myristate acetate (PMA) for 48 h. Then, *F. novicida* strains were added at a multiplicity of infection (MOI) = 1. Next, plates were centrifuged for 10 min at 300 × *g* and incubated for 1 h at 37°C. The cells were washed three times with RPMI 1640 medium, and extracellular bacteria were killed with gentamicin at 50 μg/ml for 1 h. The cells were then incubated in fresh medium at 37°C for the times indicated. To measure intracellular growth, the cells were washed with PBS and then lysed with 0.1% Triton X-100 in CDM. The number of CFUs was determined on BHIc agar plates *via* plating serial dilutions of cultures. Similar methods were used to determine growth in BmN4 cells (3 × 10^5^ cells/well), which were incubated in a 48-well tissue culture plate overnight at 27°C in Sf-900 III SFM medium.

### Fluorescence Microscopy

THP-1 cells (4 × 10^5^ cells/well) were incubated with 200 nM PMA for 48 h on 12 mm glass coverslips in 24-well tissue culture plates. GFP-expressing *F. novicida* strains were introduced at MOI = 1. Plates were centrifuged for 10 min at 300 × *g* and incubated for 1 h at 37°C. The cells were washed three times with RPMI 1640 medium, and extracellular bacteria were eliminated with gentamicin at 50 μg/ml for 1 h. The cells were then incubated in fresh medium at 37°C for the times indicated. Cells were fixed with 4% paraformaldehyde at room temperature for 30 min. A FluoView FV100 confocal laser scanning microscope (Olympus, Tokyo, Japan) was used to obtain images of the cells. Similarly, BmN4 cells (3 × 10^5^ cells/well) were grown on 12 mm glass coverslips in Sf-900 III SFM in 24-well tissue culture plates and were incubated overnight at 27°C; a similar procedure was performed. To examine the hemocytes, the hemocoels of day 2 fifth instar larvae were inoculated with 50 μl of bacterial suspension at 5 × 10^6^ CFU/larva. At indicated times after inoculation, hemolymph was collected *via* an incision in the caudal horn and observed using a FluoView FV100 confocal laser scanning microscope.

### Trypan Blue Exclusion Assay

BmN4 cells (3 × 10^5^ cells/well) were incubated in a 24-well tissue culture plate overnight and were then infected with *F. novicida* strains at MOI = 1. The plates were then centrifuged for 10 min at 300 × *g* and incubated for 1 h. Subsequently, the cells were washed three times with Sf-900 III SFM medium, and extracellular bacteria were eliminated with gentamicin at 50 μg/ml for 1 h. Cells were provided with fresh medium and grown for 48 h at 27°C. Cells were then resuspended in an equivalent volume of 0.5% trypan blue solution (Nacalai Tesque, Kyoto, Japan) to facilitate counting of live (trypan blue negative) or dead (trypan blue positive) cells using a hemocytometer. Cell death was determined as the percentage of dead (blue) cells in the grid square.

### Lactate Dehydrogenase (LDH) Release Assay

THP-1 cells (4 × 10^5^ cells/well) were incubated in a 48-well tissue culture plate with 200 nM PMA for 48 h. Cells were infected with *F. novicida* strains at MOI = 0.01. The plates were centrifuged for 10 min at 300 × g and incubated for 1 h and then washed three times with RPMI 1640 medium, and extracellular bacteria were eliminated with gentamicin (50 μg/ml for 1 h). Cells were then incubated in fresh medium at 37°C for 48 h. Release of LDH into culture medium was measured using an LDH Cytotoxicity Detection Kit (Takara Bio).

### Immunoblotting

Hemocoels of day 2 fifth instar larvae were inoculated with 50 μl of bacterial suspension at OD_595_ = 0.05 in BHIc medium using a 1 ml syringe equipped with a 30-gauge needle. After 6, 12, and 24 h, the hemolymph was collected from the caudal horn. The proteins in 0.5 μl of hemolymph were separated by SDS-PAGE on a 4%–12% Bis-Tris Gel (Thermo Fisher) and were transferred onto polyvinylidene difluoride membranes (Millipore, Billerica, MA, USA). The membranes were incubated overnight with anti-cecropin B antibody (1:1,000; ab27571; Abcam, Cambridge, UK) at 4°C. The membrane was then incubated with horseradish peroxidase-conjugated secondary antibody (0.01 μg/ml) at room temperature and was developed using an enhanced chemiluminescence detection system (GE Healthcare Life Science, Little Chalfont, UK).

### Statistical Analysis

Multiple comparisons using the Tukey–Kramer test were used to evaluate the significance of differences between groups. The Kaplan–Meier method was used to generate survival curves, and the log-rank test was used to evaluate the significance of differences compared with the wild type strain; *P* < 0.05 indicates a significant difference.

## Results

### Identification of Genes Required for the Growth of *F*. *novicida* in Arthropods

**Figure 1:**
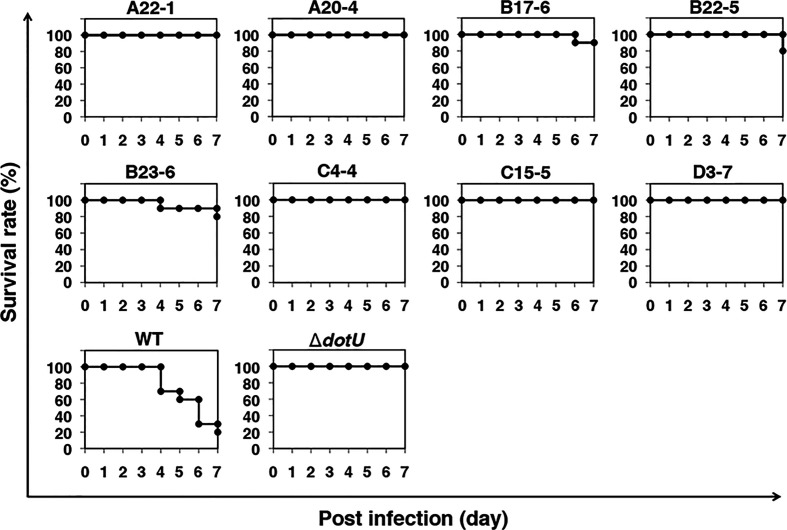
Screening of a transposon mutant library using a silkworm infection model. Each group of silkworms (n = 10) was infected with either the wild-type (WT), negative control (Δ*dotU*), or transposon mutant strains of *F. novicida* at 5 × 10^6^ CFU/larva. Survival was determined at each time point as indicated.

We previously demonstrated that *F*. *novicida* can replicate in silkworms and ultimately promote death (Saha et al., 2017). To identify novel factors that support growth of *F*. *novicida* in arthropods, silkworms were infected with a transposon mutant library previously developed (Nakamura et al., 2019); strains with reduced pathogenicity for silkworms were identified. The screening was performed by infecting three silkworms with each transposon mutant of *F*. *novicida*. Among 750 characterized transposon mutants, we identified 26 mutants that failed to kill at least one of the three silkworms; these were identified as strains with reduced pathogenicity. To confirm the reduced pathogenicity of these mutants, 10 silkworms were infected with each of these 26 transposon mutants. A Δ*dotU* strain, lacking a gene encoding part of the type VI secretion system, was used as a negative control (Suzuki et al., 2016). Among the 26 mutants, we identified 8 mutants that permitted more than 80% survival; these were identified as strains with minimal pathogenicity in the silkworm infection model (**Figure 1**). To identify the genes responsible for the pathogenicity, transposon insertion sites of the mutant strains were evaluated; seven unique genes were identified (**Table 1**). In this study, we focused on the characteristics and pathogenicity associated with *mltA* (FTN_1286), the gene encoding hypothetical membrane-bound lytic transglycosylase MltA.

**Table 1:** Sequence analysis of transposon mutants.

Strain	Locus_tag	Gene name	Product
A20-4	FTN_0496	*slt*	Soluble lytic murein transglycosylase
A22-1	FTN_0420	*purD*	SAICAR synthetase/phosphoribosylamine-glycine ligase
B17-6	FTN_1323	*iglB*	Intracellular growth locus protein B
B22-5	FTN_1641	*ampG*	MFS transport protein
B23-6	FTN_1641	*ampG*	MFS transport protein
C4-4	FTN_0177	*purH*	AICAR transformylase/IMP cyclohydrolase
C15-5	FTN_1286	*mltA*	Membrane-bound lytic murein transglycosylase
D3-7	FTN_1159	*ggt*	Gamma-glutamyl transpeptidase

### Characteristics of an *mltA* Deletion Mutant

**Figure 2:**
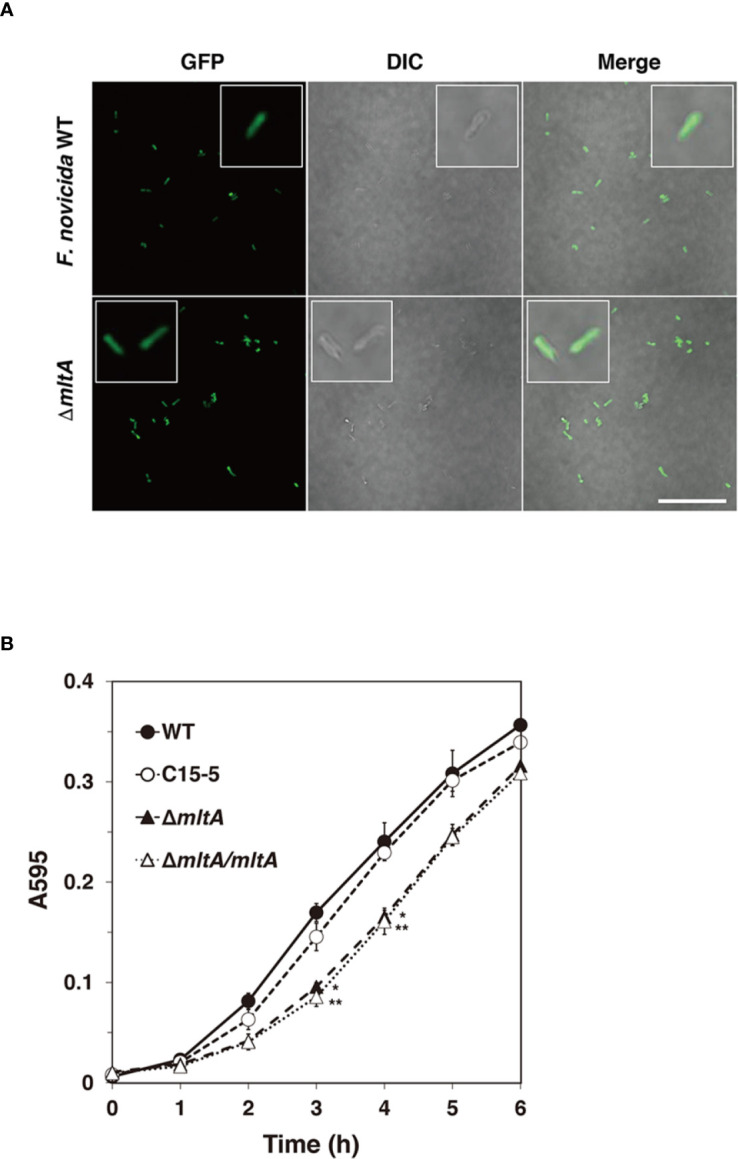
Characteristics of the *mltA* deletion mutant. **(A)** The green fluorescent protein (GFP)-expressing wild-type (WT) and Δ*mltA* strains of *F. novicida* at early-exponential phase in BHIc medium. High-magnification images are as shown in the box frame. Scale bar = 50 µm. **(B)** The wild-type (WT), transposon mutant (C15-5), Δ*mltA*, and the complemented (Δ*mltA/mltA*) strains of *F. novicida* were cultured in BHIc medium. Absorbance was measured at 595 nm. The data represent the averages and standard error of three identical experiments. Differences from the wild-type strain were determined *via* multiple comparison methods and are indicated by asterisks; **P < 0.01, *P < 0.05.

To analyze the effect of MltA in *Francisella* infection, a Δ*mltA* strain of *F*. *novicida* was constructed *via* homologous recombination. The known functions of MltA are related to degradation of cell wall peptidoglycan (PG); cell morphology and growth rates of the mutant strains were evaluated. The Δ*mltA* cells all maintained the characteristic rod shape in the early-exponential phase of liquid culture and were otherwise indistinguishable from the wild-type strain (**Figure 2A**). The growth rates of Δ*mltA* strain and of a complemented (Δ*mltA*/*mltA*) strain in liquid culture medium were slightly slower than those of the wild-type strain and of the transposon mutant at mid-exponential phase; there were no significant differences at late- exponential phase (**Figure 2B**).

### Role of MltA in Promoting Multiplication and Virulence of *F*. *novicida* in Silkworm

**Figure 3:**
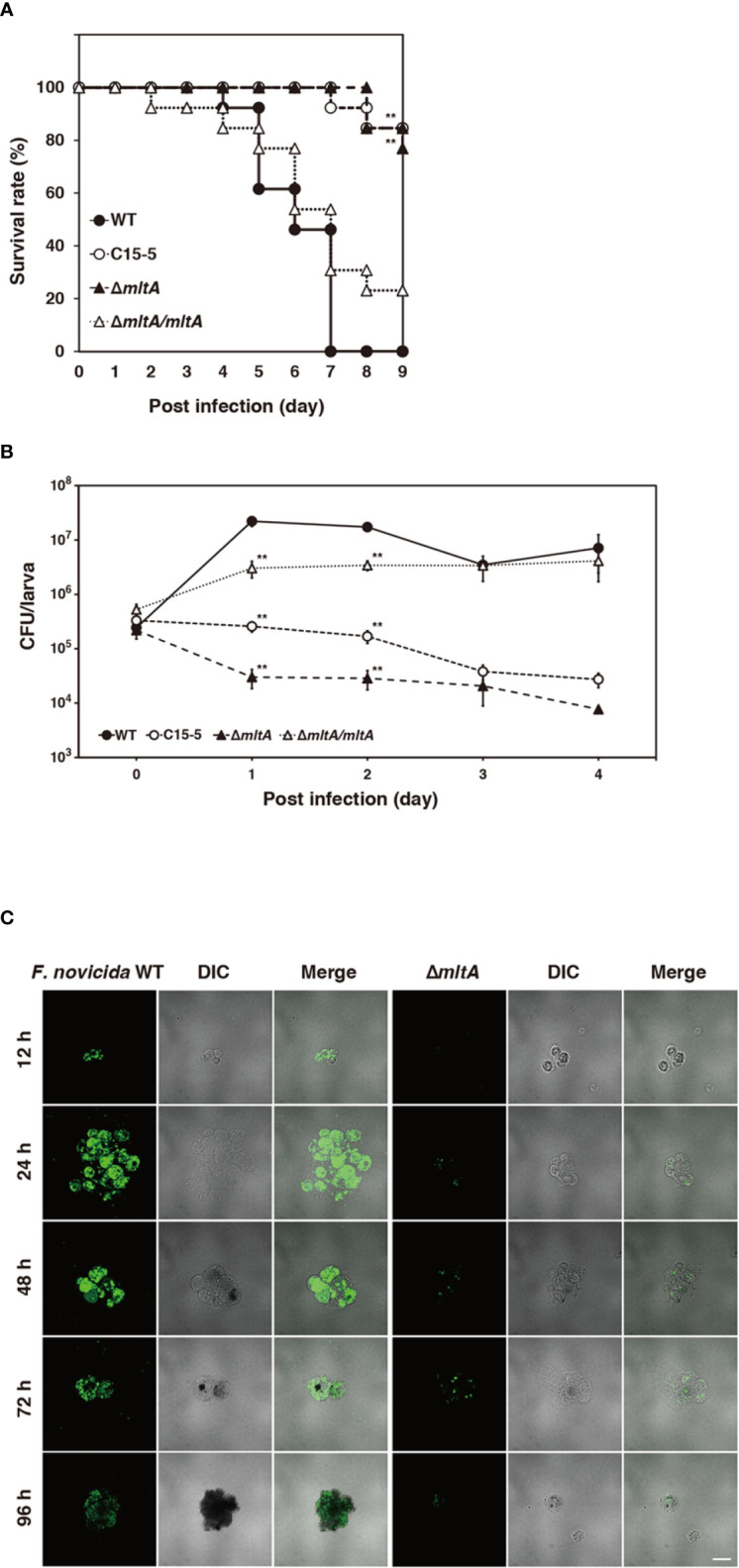
Role of MltA in multiplication and virulence of *F. novicida* in the silkworm infection model. **(A)** Each group of silkworms (n = 13) was infected with the wild-type (WT), transposon mutant (C15-5), Δ*mltA*, or the complemented (Δ*mltA/mltA*) strains of *F. novicida* at 5 × 10^6^ CFU/larva. Survival was evaluated at the time points; differences from the wild-type strain were evaluated *via* log-rank test; **P < 0.01. **(B)** Each group of silkworms (n = 15) was infected with the wild-type (WT), transposon mutant (C15-5), Δ*mltA*, and the complemented (Δ*mltA/mltA*) strains of *F. novicida* at 5 × 10^6^ CFU/larva. Bacterial numbers in silkworms were determined at 0 (immediately after infection), 1, 2, 3, 4 days post infection. These data represent the averages and standard error of bacterial numbers in three different silkworms. Differences from the wild-type strain were determined *via* multiple comparisons and are indicated by asterisks; **P < 0.01. **(C)** Silkworms were infected with the green fluorescent protein (GFP)-expressing wild-type (WT) or Δ*mltA* strains of *F. novicida*. Hemolymph was harvested at time points indicated and observed by confocal laser scanning microscopy. Scale bar = 20 µm.

To explore the role of *mltA* in promoting virulence, silkworms were infected with the Δ*mltA* strain. All silkworms infected with the wild-type strain were dead within 7 days; by contrast, 80%–90% of the silkworms infected with the Δ*mltA* or transposon mutant strains remained viable at 9 days after infection (**Figure 3A**). Complementation (Δ*mltA*/*mltA*) restored virulence; survival of silkworms infected with this strain was comparable to that of the wild-type strain (**Figure 3A**). The number of bacterial cells in whole silkworms was also determined. As expected, the wild-type and complemented strains increased in number through 1 to 2 days post infection in whole silkworms; no growth of the Δ*mltA* or transposon mutant strains was detected (**Figure 3B**). A similar tendency was observed when analyzing the silkworm hemolymph. Specifically, when silkworms were infected with a GFP-expressing wild-type *F. novicida*, the bacterial population detected in the hemolymph increased at 24 to 48 h post infection (**Figure 3C**). This was not observed in response to infection with GFP-expressing Δ*mltA* strain (**Figure 3C**). Taken together, these results indicate that MltA is important for growth and virulence of *F. novicida* in silkworms.

### Effect of MltA on Intracellular Growth and Cytotoxicity of *F*. *novicida* in BmN4 Cells

**Figure 4:**
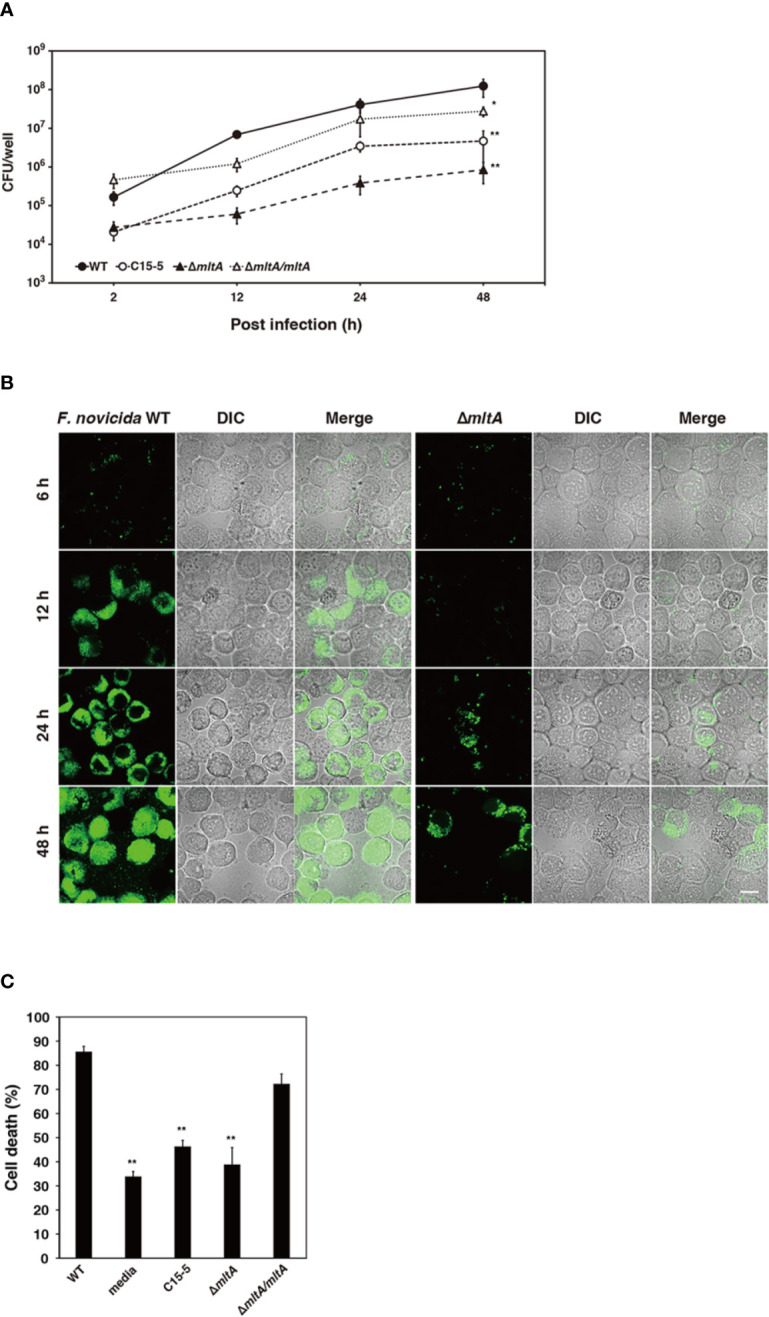
Impact of MltA on intracellular growth and cytotoxicity of *F. novicida* in BmN4 cells. **(A)** BmN4 cells were infected with the wild-type (WT), transposon mutant (C15-5), Δ*mltA*, or the complemented (Δ*mltA/mltA*) strains of *F. novicida* at MOI = 1 followed by treatment with 50 µg/ml gentamicin for 1 h. The cells were disrupted with 0.1% Triton X-100 and plated on BHIc agar at the time points indicated. **(B)** BmN4 cells were infected with the green fluorescent protein (GFP)-expressing wild-type (WT) or Δ*mltA* strains of *F. novicida* at MOI = 1 and treated 50 µg/ml gentamicin for 1 h. The cells were fixed and evaluated by confocal laser scanning microscopy at indicated time points. Scale bar = 20 µm. **(C)** BmN4 cells were infected with the wild-type (WT), transposon mutant (C15-5), Δ*mltA*, and the complemented (Δ*mltA/mltA*) strains of *F. novicida* at MOI = 1 and treated 50 µg/ml gentamicin for 1 h and incubated for 48 h. Cell survival was evaluated by trypan blue exclusion assay. **(A, C)** The data represent the averages and standard error of three identical experiments. Differences from the wild-type strain were determined *via* multiple comparisons and are indicated by asterisks; **P < 0.01, *P < 0.05.

To confirm the role of MltA in promoting intracellular growth of *F*. *novicida* in silkworm cells, *F. novicida* strains were used to infect to cultures of the silkworm ovary BmN4 cell line (Grace, 1967). The wild-type strain grew intracellularly in BmN4 cells from 2 to 48 h post infection; by contrast, the Δ*mltA* and transposon mutant strains showed comparatively limited growth under these conditions. The complemented strain restored the capacity for intracellular growth (**Figures 4A, B**). To investigate a role for MltA in promoting cytotoxicity, BmN4 cells were infected with *F. novicida* strains and cell death was evaluated by the trypan blue exclusion assay. Among our results, the Δ*mltA* strain was significantly less cytotoxic than either the wild-type or complemented strains (**Figure 4C**). These results suggest that MltA is necessary for intracellular growth and is a critical factor underlying the cytotoxicity of *F*. *novicida* within silkworm cells.

### Immune Suppression of Silkworm Promoted by MltA

**Figure 5:**
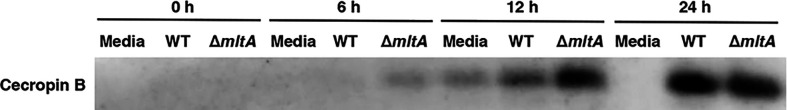
MltA-mediated host immune suppression in the silkworm infection model. Hemolymph plasma samples from silkworms infected with the wild-type (WT) or the Δ*mltA* strains of *F. novicida* were collected at post-infection time points as indicated. Cecropin B expression was determined by immunoblotting. Representative data from three independent experiments are as shown. Full blots are included in **Supplementary Figure 1**.

*F*. *tularensis* suppresses host immune systems of both mammals and arthropods (Suzuki et al., 2016; Putzova et al., 2017). One of the main immune responses of arthropods when confronted with pathogens is the production of antimicrobial peptides (AMPs) (Chen and Lu, 2018). To explore the impact of MltA on the immune responses in silkworms, we examined expression levels of the major AMP, cecropin B, by immunoblotting. We found that the induction of cecropin B expression was accelerated in response to infection with the Δ*mltA* strain. The Δ*mltA*-induced expression of cecropin B was detected as early as 6 h post infection, and no response was observed in response to infection with the wild-type strain at this time point. Cecropin B was detected at 12 h post infection with both the wild-type and Δ*mltA* strains, although expression was stronger in the Δ*mltA*-infected silkworms. At 24 h post infection, expression levels were nearly indistinguishable (**Figure 5**). These results suggest that *F*. *novicida* promotes host immune suppression at the early stages of infection *via* an MltA-dependent mechanism.

### Impact of MltA on the Pathogenicity of *F. novicida* in Human Macrophage Cell Line

**Figure 6:**
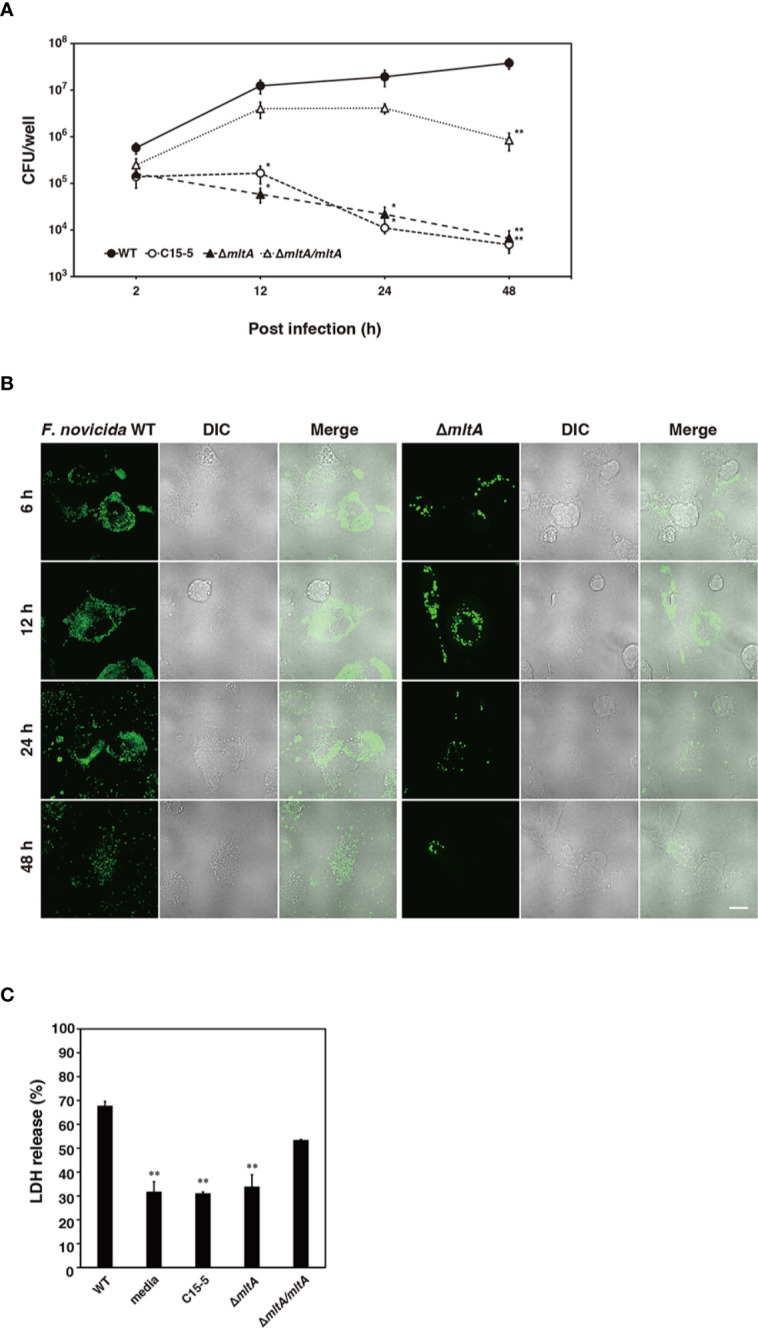
Impact of MltA on pathogenicity of *F. novicida* in a human macrophage cell line. **(A)** THP-1 cells were infected with the wild-type (WT), transposon mutant (C15-5), Δ*mltA*, or the complemented (Δ*mltA/mltA*) strains of *F. novicida* at multiplicity of infection (MOI) = 1 and treated 50 µg/ml gentamicin for 1 h. Cells were disrupted with 0.1% Triton X-100 and plated on BHIc agar at the times indicated. **(B)** THP-1 cells were infected with the green fluorescent protein (GFP)-expressing wild-type (WT) or Δ*mltA* strains of *F. novicida* at MOI = 1 and treated 50 µg/ml gentamicin for 1 h. Cells were fixed and evaluated by confocal laser scanning microscopy at the time points as indicated. Scale bar = 20 µm. **(C)** THP-1 cells were infected with the wild-type (WT), transposon mutant (C15-5), Δ*mltA*, or the complemented (Δ*mltA/mltA*) strains of *F. novicida* at MOI = 0.01 and treated 50 µg/ml gentamicin for 1 h. Cells were incubated for 48 h and cytotoxicity following infection was measured using the lactate dehydrogenase (LDH) assay. **(A, C)** The data represent the averages and standard error of three identical experiments. Differences from the wild type strain were evaluated *via* multiple comparisons and are indicated by asterisks; **P < 0.01, *P < 0.05.

Intracellular growth of *Francisella* in macrophages is closely related to its pathogenicity in humans (Ozanic et al., 2015). To determine whether *mltA* plays an important role in human virulence, cells of the human monocyte cell line, THP-1, were infected with the wild-type and mutant strains of *F. novicida*. The wild-type strain multiplied intracellularly within THP-1 cells at 2 to 48 h post infection (**Figures 6A, B**). The Δ*mltA* and transposon mutant strains failed to grow in THP-1 cell culture; their numbers decreased during this time period (**Figures 6A, B**). Complementation restored the capacity for intracellular growth during 2 to 24 h post infection. To explore the role of MltA in promoting cytotoxicity in human macrophages, THP-1 cells were infected with *F. novicida* strains and cytotoxicity was measured using the LDH release assay. Infection with the Δ*mltA* and transposon mutant strains showed less cytotoxicity than that with the wild-type and complemented strains (**Figure 6C**). These results suggest that MltA is essential for intracellular growth of *F. novicida* in human macrophages.

## Discussion

*Francisella* is transmitted to mammalian hosts by arthropod vectors (Petersen et al., 2009); as such, a careful analysis of the unique growth mechanisms in arthropods is important for our understanding of the full life cycle of *Francisella*. In this study, we identified a novel factor, which promotes growth and survival of *F*. *novicida* in arthropods using the silkworm infection model. We previously developed two distinct silkworm infection models. The first uses LVS. In this infection model, LVS was maintained and the infected silkworms survived, resulting in symbiotic relationship (Suzuki et al., 2016). The second features *F. novicida*; *F*. *novicida* proliferates within the silkworms and ultimately promotes their death (Saha et al., 2017). As bacterial symbiosis and growth within arthropods are considered to be related processes in the *Francisella* life cycle, symbiotic factors that predominate in LVS infection and growth factors associated with *F. novicida* infection may ultimately cooperate to promote survival of *Francisella* in arthropods. We identified herein critical factors underlying bacterial growth in the *F. novicida*–silkworm infection model; specifically, we identified seven distinct genes that were critical for *F. novicida* growth in silkworms by screening a transposon mutant library.

To date, arthropod infection models, such as wax moth (*G. mellonella*) and fruit fly (*D*. *melanogaster*), have been used to explore features of the *Francisella*–arthropod interaction (Aperis et al., 2007; Vonkavaara et al., 2008; Ahlund et al., 2010; Moule et al., 2010). Several of the aforementioned seven genes (*iglB*, *slt*) identified based on screening using the silkworm infection model include those previously identified in *G. mellonella* or *D*. *melanogaster* infection models (Ahlund et al., 2010; Vonkavaara et al., 2013). Several of these genes have already been reported to encode pathogenic factors related to *Francisella* infection in humans. Despite the previous screenings, MltA encoded by *mltA* had not been reported until now as a pathogenic factor in human infection or a bacterial growth factor in arthropods. Although many reports point to bacterial-derived lytic transglycosylases (LTs) as closely associated with pathogenicity (Koraimann, 2003), information about the function of LTs associated with *Francisella* infection in mammals or arthropods is limited (Bachert et al., 2019; Nakamura et al., 2019). Given these previous findings, we proceeded on the assumption that *mltA* might be involved in proliferation of *F. novicida* in arthropods and that this function might be revealed in our silkworm infection model.

To analyze functions of MltA in *F. novicida*, we generated a Δ*mltA* strain. Infection with the wild-type strain killed silkworms, while that with the Δ*mltA* or associated transposon mutant strains permitted longer-term survival. Intracellular growth of the Δ*mltA* and transposon mutant strains was reduced in lymphocyte and in the silkworm-derived cell line BmN4 when compared with growth of the wild-type and complemented strains. These results indicate that MltA is a critical factor and is required to support intracellular growth in silkworms. In addition, bacterial numbers of Δ*mltA* and transposon mutant strains in BmN4 cells at early stage of infection (2 h post infection) tended to be decreased compared with that of the wild-type and complemented strains, indicating that MltA of *F. novicida* may affect the invasion ability to silkworm cells.

LTs are known to be involved in cell division and membrane stability of other bacteria (Koraimann, 2003); as such, we considered the possibility that the Δ*mltA* and/or the transposon mutant strains might exhibit growth defects in the culture medium. Bobie et al. (Adu-Bobie et al., 2004) reported that a Δ*mltA* strain of *Neisseria meningitidis* exhibited delayed growth *in vitro*. In our study, we found that the Δ*mltA* strain showed similar morphology to the wild-type strain but grew at slightly decreased rates in liquid culture medium. Interestingly, the transposon mutant exhibited no growth delay, and *mltA* complementation failed to restore the growth rate. These data suggest that growth delay may be a structural result associated with the gene deletion. For example, the deletion of the *mltA* gene may have an impact on the expression of neighboring genes. Of note, although growth of Δ*mltA* strain was only slightly delayed in liquid culture, its growth was significantly inhibited in silkworms and in cultures of BmN4 and THP-1 cells. Likewise, while growth of the transposon mutant was similar to that of the wild-type strain in liquid culture, its growth in silkworms, BmN4 cells, and THP-1 cells was markedly reduced. Moreover, despite the slightly delayed growth of the complemented strain when evaluated in liquid culture, complementation restored the capacity for growth in silkworms, BmN4 cells, and THP-1 cells. From these results, we concluded that diminished proliferation of the Δ*mltA* strain in both silkworms and cell lines could not be attributed to factors promoting growth delay in liquid culture.

MltA is a membrane-bound lytic murein transglycosylase, which belongs to the LT enzyme family. LTs show same substrate specificity as the muramidases (lysozymes), and catalyze the cleavage of β-1,4-glycosidic bond between N-acetylglucosamine and N-acetylmuramic acid residues of PG, but resulted in the different formation of product with 1,6-anhydromuramyl residues (Holtje et al., 1975). LTs are lytic enzymes that target PG and promote appropriate cell division and membrane stability (Dik et al., 2017). LTs also create spaces within the PG to facilitate assembly of macromolecular complexes including flagella, pili, and secretion systems larger than the size of PG pores (Scheurwater et al., 2008). Specifically, LTs were associated with the activity of the type IV secretion system in both *Brucella abortus* and *Helicobacter pylori* (Rohde et al., 2003; Bao et al., 2017) and with the type VI secretion system of *Escherichia coli* (Santin and Cascales, 2017). *Francisella* spp. have a type VI secretion system, which plays an important role in promoting growth within arthropods; this secretion system was previously reported to be critical for intracellular growth in a mosquito cell line (Read et al., 2008). In addition, we previously showed that the type VI secretion system was directly involved in proliferation of *Francisella* within silkworms (Saha et al., 2017). In *Acinetobacter*, soluble lytic transglycosylase (SLT) is required in order to form type IV pili, which are associated with bacterial pathogenicity (Crepin et al., 2018). In *F. novicida*, peptidase and chitin-binding proteins are secreted from the type IV pili apparatus (Hager et al., 2006). These findings suggest that proteins secreted from type VI secretion system or type IV pili may contribute to the intracellular growth and/or to the immunosuppressive activity associated with *F. novicida* infection.

While we could not fully clarify the detailed mechanisms *via* which *F*. *novicida* MltA promotes growth and survival in silkworms, our results suggest a critical role played by its capacity to suppress host immune responses. Previous studies have reported that *Francisella* spp. are generally capable of suppressing host immune responses, thus enabling them to survive within the host (Jones et al., 2012). Our previous studies in silkworms showed that both *F. novicida* and LVS were capable of inhibiting host immune activity (Suzuki et al., 2016; Saha et al., 2017). Arthropods, including silkworms, possess Toll pathways to overcome the pathogen infection (Cheng et al., 2008; Kurata, 2014). Pathogen-associated molecular pattern molecules, including lipopolysaccharide (LPS) and PG, were captured by recognition proteins, resulting in immune responses that include expression of AMPs (Chen and Lu, 2018). Cecropin B is one of major AMPs effective against both Gram-positive and Gram-negative bacteria (Moore et al., 1996); cecropin B was shown to be effective against *F*. *novicida* in a *D*. *melanogaster* infection model (Vonkavaara et al., 2013). In this study, expression of cecropin B was suppressed during the early stages, at 6–12 h post infection with the wild-type strain. By contrast, although the bacterial number was diminished, the Δ*mltA* strain induced high levels of expression of cecropin B compared to that observed in response to infection with the wild-type strain. These results suggest that limited survival of the Δ*mltA* strain may be attributed to the fact that it does not suppress expression of AMPs in infected silkworms. MltA has been associated with PG remodeling, LPS synthesis, and the production of outer membrane vesicles (OMVs) in various bacterial species (Ferrari et al., 2006; Suvorov et al., 2008; Liu et al., 2012). In *Neisseria gonorrhoeae*, LTs plays an important role in escape of the bacterium from TLR recognition *via* control of OMVs production (Knilans et al., 2017). These findings in earlier works may suggest that MltA of *Francisella* modulates exposure of LPS, PG, and OMVs and thus avoids host recognition by modifying membrane structures.

In this study, MltA was identified as a growth factor in the silkworm infection model. MltA also contributed to intracellular growth and cytotoxicity in human macrophages. Among the seven genes identified as growth factors in silkworms, four genes encoding the major facilitator superfamily (MFS) transport protein, intracellular growth locus protein B, gamma-glutamyl transpeptidase, and SLT were previously identified as promoting pathogenicity in mammalian hosts (Bröms et al., 2010; Ireland et al., 2011; Balzano et al., 2018). These results suggest that many of the factors that promote growth and survival in silkworms may also be involved in intracellular proliferation in mammal cells. This finding is consistent with the suggestion made by Akimana and Kwaik (2011), specifically that the mechanisms associated with the *Francisella* pathogenicity may depend on systems that are common to or at least similar among arthropods and mammals. As such, we conclude that the *F. novicida*–silkworm infection model is useful as an infection model for mammals, including humans.

In conclusion, our research has highlighted the importance of MltA of *F. novicida* as a novel factor that promotes bacterial growth in silkworms and similarly contributes to pathogenicity in human cells. Because highly pathogenic *F. tularensis* subspecies possess *mltA*, it might be a potential target for the control of arthropod-mediated transmission of *F. tularensis*.

## Data Availability Statement

The raw data supporting the conclusions of this article will be made available by the authors, without undue reservation.

## Author Contributions

TN contributed to laboratory analysis, investigation, and writing original draft. TS contributed to conceptualization, laboratory analysis, investigation, and writing original draft. FI, SO, and SS contributed to laboratory analysis. AU contributed to methodology and resources. KW contributed to laboratory analysis, investigation, and validation. MW was involved in conceptualization, laboratory analysis, supervision, review, and editing. All authors contributed to the article and approved the submitted version.

## Funding

This work was supported by JSPS KAKENHI Grant Number 15K08463, 17H03914, and 19K07556.

## Conflict of Interest

The authors declare that the research was conducted in the absence of any commercial or financial relationships that could be construed as a potential conflict of interest.
