# Supplementary material for: Identification of Membrane-Bound Lytic Murein Transglycosylase A (MltA) as a Growth Factor for Francisella novicida in a Silkworm Infection Model
Source: Front Cell Infect Microbiol. 2021 Jan 22;10:581864. doi: 10.3389/fcimb.2020.581864 (PMC7862118; doi:10.3389/fcimb.2020.581864)
Supplement: Supplementary file 1 [file DataSheet_1.docx]

Supplementary Table 1. Primer sets and templates.

Supplementary Figure 1. Immune suppression of silkworm through MLTA. Hemolymph plasma samples from silkworms infected with wild-type (WT) and Δ*mltA* of *F. novicida* were collected at indicated time post infection. Cecropin B expression levels were analyzed by immunoblotting.
